# The Effects of Methylphenidate (Ritalin) on the Neurophysiology of the Monkey Caudal Prefrontal Cortex

**DOI:** 10.1523/ENEURO.0371-18.2018

**Published:** 2019-03-04

**Authors:** Sébastien Tremblay, Florian Pieper, Adam Sachs, Ridha Joober, Julio Martinez-Trujillo

**Affiliations:** 1 Montreal Neurological Institute, McGill University, Montreal, Québec, H3A 2B4, Canada; 2 Institute for Neurophysiology and Pathophysiology, University Medical Center Hamburg-Eppendorf 20251, Hamburg, Germany; 3 Department of Surgery, The Ottawa Hospital Research Institute, University of Ottawa, Ottawa, Ontario, K1H 8L6, Canada; 4 Douglas Mental Health University Institute, McGill University, Montreal, Québec, H4H 1R3, Canada; 5 Robarts Research Institute, Departments of Psychiatry, Physiology and Pharmacology; 6 Schulich School of Medicine and Dentistry, Brain and Mind Institute, University of Western Ontario, London, Ontario, N6A 5C1, Canada

**Keywords:** attention, methylphenidate, multielectrode array, prefrontal cortex, primates, Ritalin

## Abstract

Methylphenidate (MPH), commonly known as Ritalin, is the most widely prescribed drug worldwide to treat patients with attention deficit disorders. Although MPH is thought to modulate catecholamine neurotransmission in the brain, it remains unclear how these neurochemical effects influence neuronal activity and lead to attentional enhancements. Studies in rodents overwhelmingly point to the lateral prefrontal cortex (LPFC) as a main site of action of MPH. To understand the mechanism of action of MPH in a primate brain, we recorded the responses of neuronal populations using chronic multielectrode arrays implanted in the caudal LPFC of two macaque monkeys while the animals performed an attention task (*N* = 2811 neuronal recordings). Over different recording sessions (*N* = 55), we orally administered either various doses of MPH or a placebo to the animals. Behavioral analyses revealed positive effects of MPH on task performance at specific doses. However, analyses of individual neurons activity, noise correlations, and neuronal ensemble activity using machine learning algorithms revealed no effects of MPH. Our results suggest that the positive behavioral effects of MPH observed in primates (including humans) may not be mediated by changes in the activity of caudal LPFC neurons. MPH may enhance cognitive performance by modulating neuronal activity in other regions of the attentional network in the primate brain.

## Significance Statement

Methylphenidate (MPH), widely known as Ritalin, is the most prescribed drug to treat patients with attention deficits. Nonetheless, it is still unclear how and why the drug improves attention in humans. Studies in rodents point to the prefrontal cortex (PFC) as the main target of MPH. To validate these findings in primates, we trained macaque monkeys to perform an attention task while under various doses of MPH. We also chronically implanted multielectrode arrays in the posterior PFC of these monkeys to record neuronal ensemble activity during the task. Surprisingly, we found no effect of the drug on neuronal activity, even at cognitive-enhancing doses of MPH. The caudal PFC might not be the site of action of MPH in the primate brain.

## Introduction

The Centers for Disease Control and Prevention (CDC) estimate that, in the United States alone, 3.5 million children (6.1%) are taking methylphenidate (MPH), widely known as Ritalin, to circumvent the distractibility associated with attention deficit/hyperactivity disorder (ADHD; Visser et al., 2014). MPH also enhances cognitive functions in healthy humans, monkeys, and rodents (Bain et al., 2003; Arnsten and Dudley, 2005; Ilieva et al., 2015), suggesting a general mechanism of action across species. However, despite several decades of MPH being widely used in the clinic (Subcommittee on Attention-Deficit/Hyperactivity Disorder et al., 2011), we still have a limited understanding of the mechanisms by which the drug improves cognitive performance.

Neurochemical studies in rodents have revealed that MPH blocks dopamine and norepinephrine reuptake transporters at the level of synapses, modulating dopaminergic and noradrenergic receptors signaling in post-synaptic neurons (Arnsten and Dudley, 2005; Berridge et al., 2006, 2012). Although the drug is distributed across the entire nervous system after systemic administration in rodents, at low doses that improve cognitive performance, its effects appear to be localized to the prefrontal cortex (PFC; Devilbiss and Berridge, 2008; Spencer et al., 2015), a brain region that plays an instrumental role in executive functions such as selective attention and working memory (Desimone and Duncan, 1995; Miller and Cohen, 2001; Petersen and Posner, 2012). The mechanism by which an increase in catecholamine neurotransmission in PFC neuronal circuits leads to improved cognitive performance, however, remains elusive.

In rodents, pioneering work combining pharmacological interventions with single-cell electrophysiology have reported that MPH can modulate the responses of individual neurons in the PFC by increasing their selectivity for stimulus locations (Devilbiss and Berridge, 2008; Berridge and Arnsten, 2015). In primates, to our knowledge, a single study combined electrophysiology with pharmacological intervention using drugs approved for the treatment of ADHD in humans. In this study the effects of a non-stimulant drug (atomoxetine) on the spiking activity of a small sample of prefrontal neurons (*N* = 17) were investigated in a single monkey performing a working memory task (*N* = 1) using direct iontophoresis delivery to single neurons (Gamo et al., 2010). The findings of this early study were in line with what was previously found by the same investigators in the rodent, namely, an increase in the signal-to-noise ratio of persistent activity from prefrontal neurons during a working memory task. However, it is not clear whether the more clinically relevant oral administration of MPH (as opposed to iontophoresis delivery of atomoxetine) modulates the activity of populations of neurons in the primate PFC in a manner consistent with findings from basic attention research.

Over the last decades, our basic understanding of the neuronal mechanisms underlying the effects of attention on single neurons has considerably progressed (Moran and Desimone, 1985; Desimone and Duncan, 1995; Treue and Martínez Trujillo, 1999; Reynolds and Chelazzi, 2004; Lennert et al., 2011; Niebergall et al., 2011). More recently, new technologies that allow recording the activity of multiple neurons simultaneously in behaving animals (Nicolelis et al., 2003; Buzsáki, 2004) have shined a new light on those mechanisms. Notably, by using simultaneous recording techniques, two landmark studies in nonhuman primates have shown that attention improves information coding by neuronal populations primarily by reducing correlated noise between individual neurons (i.e., noise correlations) rather than modulating single neuron response (Cohen and Maunsell, 2009; Mitchell et al., 2009). In support to this finding, both theoretical (Shadlen et al., 1996; Averbeck et al., 2006; Cohen and Kohn, 2011; Moreno-Bote et al., 2014; Kanitscheider et al., 2015) and experimental (Tremblay et al., 2015b; Leavitt et al., 2017b) evidences show that noise correlations can modulate information processing in large neuronal populations. Considering these new insights from basic research, we hypothesized that MPH improves attentional processing in the PFC by recruiting similar noise reduction mechanisms.

To test this hypothesis, we trained two macaque monkeys to perform a demanding attention task that required detecting a visual target in the presence of distractors. Before different experimental sessions, we administered orally either various doses of MPH or a placebo vehicle to the monkeys. During performance of the attention task, we simultaneously recorded the responses of large neuronal populations in the caudal lateral PFC (LPFC) using chronically implanted 96-channel Utah multielectrode arrays. This region of the PFC was selected because it plays a causal role in visual attention, as demonstrated by microstimulation, pharmacological, and optogenetic studies in primates (Dias and Segraves, 1999; Moore and Fallah, 2004; Noudoost and Moore, 2011; Schafer and Moore, 2011; Acker et al., 2016). Moreover, its neurophysiological properties are very well studied and known to strongly represent attentional processing at the single neuron and neuronal ensemble levels (Buschman and Miller, 2007; Armstrong et al., 2009; Gregoriou et al., 2009, 2012; Lennert and Martinez-Trujillo, 2011; Squire et al., 2013; Tremblay et al., 2015b). In this experiment, we recorded over 55 behavioral sessions, yielding 2811 neuronal datasets from which the neuronal effects of various doses of MPH could be investigated at the single, pairwise, and neuronal ensemble levels.

## Materials and Methods

### Subjects

Two male macaque monkeys (*Macaca fascicularis*) both aged six years old and weighting 5.8 kg (monkey “F”) and 7.5 kg (monkey “JL”) participated in the experiment. All procedures complied with the Canadian National Council of Animal Care guidelines and were preapproved by the University Animal Care Committee. Over the course of a testing session, the animals would receive their daily amount of fluids as rewards for correctly performing the task. We also provided the animals with fresh fruits and vegetables as supplements when finishing a recording session. Body weight, water intake, and mental and physical hygiene were monitored on a daily basis by veterinary staff. None of the animals were sacrificed for the purpose of this study.

### Behavioral task

**Figure 1.**
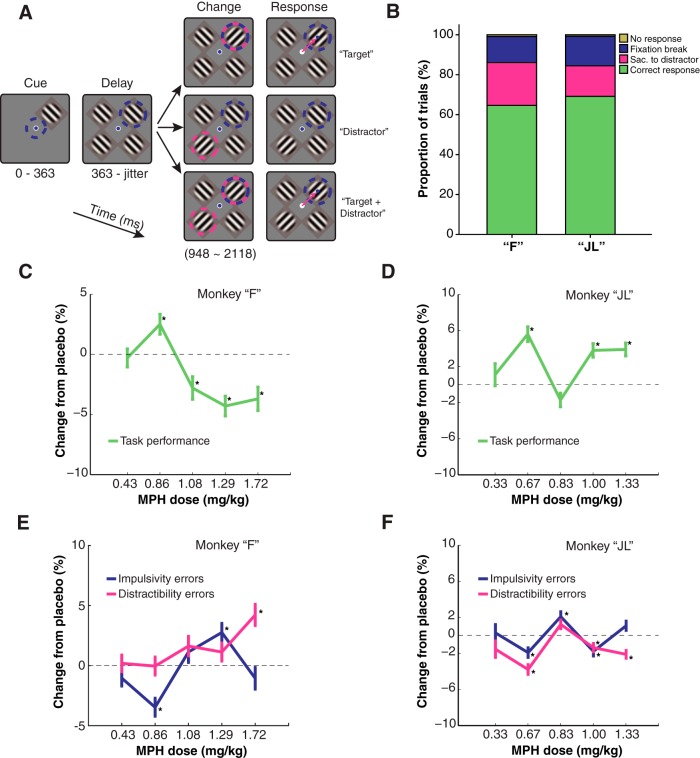
Behavioral task and performance. ***A***, Behavioral task with the three randomly interleaved trial types. Blue dashed circles represent the focus of covert attention. Pink dashed circles indicate orientation change(s). Pink arrows indicate saccadic eye movements. Blue dot represents gaze position. ***B***, Average behavioral performance of each subject under placebo sessions only. The colors indicate the proportion of each trial outcome in a behavioral session. Fixation break represents errors where the subject would respond before a Go signal was given. Sac. to distractor represents errors where the subject would respond to a distracting stimulus. No response represents trials where the subject would not provide a response. ***C***, ***D***, Line plots representing the change in overall hit rate relative to matched placebo sessions in the attention task following various doses of MPH. Hit rate is considered a proportion (Hit/Hit+Errors). Differences in proportion (hit rate) across treatment conditions are computed with χ^2^ tests. Asterisks represent statistically significant changes in hit rate relative to placebo sessions (χ^2^ test, *p* < 0.05). ***E***, ***F***, Same format as ***C***, ***D*** but representing the proportion of specific error types across treatment conditions. Up means more errors. Refer to Materials and Methods for definitions. Error bars represent the SE of the sample proportion estimate.

The monkeys were instructed to covertly sustain attention to one of four Gabor stimuli presented on a screen while ignoring the other three Gabor stimuli (distractors; Fig. 1*A*). A trial would begin with one Gabor stimulus appearing at one out of four locations on the screen for a brief period while the monkey keeps its gaze on a central fixation point (363 ms). This early Gabor stimulus was defined as the “cue,” indicating that this target had to be covertly attended to during the entire trial (while keeping gaze on the central fixation point). After the cue presentation, three other Gabor stimuli would appear on the screen at the three remaining locations. A variable delay period would follow (585–1755 ms). Three different trial types were randomly interleaved within a session. In “target” trials, after a variable delay interval, the target Gabor quickly changed orientation (90° clockwise rotation) indicating the monkey to saccade toward the target location to earn a juice reward (250-ms response time window). In “distractor” trials, the orientation change occurred in the distractor Gabor opposite to the target location. To earn a reward on those trials, the monkey had to inhibit saccading to the distracting Gabor and maintain fixation on the central dot. In “target + distractor” trials, two simultaneous orientation changes co-occurred in the target Gabor and in the distractor opposite to the target. The monkeys had to make a saccade toward the target and not toward the distractor to earn the reward. Every trial was divided into three time epochs: (1) the cue epoch (cue onset to 200-ms postcue onset); (2) the attention epoch (600-ms postcue onset to 1000-ms postcue onset); (3) the saccade epoch (50 ms before to 50 ms after saccade onset). The monkeys’ gaze position was monitored at a rate of 500 Hz using an infrared video-based eye-tracking system (Eyelink 1000, SR Research). Monkey “F” completed a mean (STD) of 817.22 (93.43) trials per session. Monkey “JL” completed an average of 715.00 (100.44) trials per session. The average length of a session for monkey “F” was 2.26 (0.28) h, and 1.44 (0.19) h for monkey “JL.”

Our subjects could make several different types of errors while performing this attention task, which can be broadly related to different types of maladapted behaviors in humans. For one, monkeys could erroneously break fixation during the cue or the delay epoch, that is, before a Go signal (the change in orientation) is presented. This error type could loosely be related to impulsivity, that is, the propensity to respond prematurely without foresight (Winstanley et al., 2006). A second error type noticeable in our behavioral task is the propensity to respond to a distractor Go signal. For example, monkeys would sometime saccade to the distractor location on a change in the distractor stimulus orientation that ought to be ignored to successfully complete the trial. We can loosely relate this error type to the concept of distractibility in humans, which is the propensity to pay attention to stimuli irrelevant for the task at hand. These two error types, impulsivity and distractibility, will be analyzed for each drug dose in addition to overall task performance. Finally, a general indicator of motivation while be inferred from the total number of trials completed by the animals in a given session. Motivation has also been shown to be influenced by MPH in some studies with nonhuman primates (Rajala et al., 2012). Nowhere in this study will we pretend that our experiment offers an “animal model” of ADHD, impulsivity, or distractibility. The terms “impulsivity” and “distractibility” are used without direct connection to the symptomatology of ADHD in humans.

### Surgical procedure

**Figure 2.**
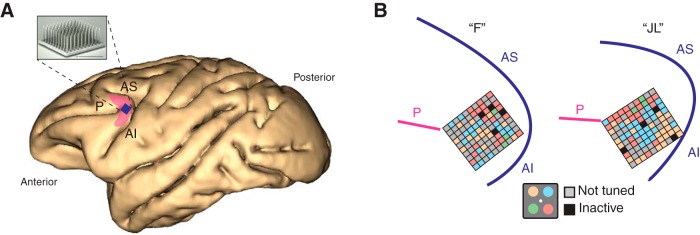
Neurophysiological recordings. ***A***, Location of chronically implanted multielectrode Utah array within the left caudal LPFC. The shaded pink area roughly represents area 8A in the macaque brain. The blue square represents implant location. P: principal sulcus. AS: arcuate sulcus superior. AI: arcuate sulcus inferior. ***B***, Implant location based on intra-operative photography for both monkey “F” and monkey “JL” in reference to major sulci. Each small square represents one of the 96 microelectrodes on the array. Colors represent the spatial attentional tuning of the neurons recorded at each electrode site as a function of the four quadrant locations (inset). Note tuned stands for neurons that do not show attentional modulation. Inactive represents reference electrodes and grounds.

Surgeries were conducted under general anesthesia using isofluorane administered via endotracheal intubation. Previous to the neuronal recordings the animals were implanted with titanium head posts used to restrain head motion and allow accurate measures of eye movements during training and recording sessions. We chronically implanted 96-channel “Utah” multielectrode arrays (Blackrock Microsystems) in each monkey’s left caudal LPFC following a surgical procedure described elsewhere (Fig. 2*A*; Leavitt et al., 2013). The multielectrode array was inserted on the prearcuate convexity posterior to the caudal end of the principal sulcus and anterior to the arcuate sulcus, a cytoarchitectonic region known as area 8A, the homolog of area 8A in the human LPFC (Fig. 2*A*,*B*; Petrides and Pandya, 1999; Petrides, 2005). The array connector was fixed to the skull using titanium screws and bone cement providing easy access during recording sessions.

### Neurophysiology

We simultaneously recorded the spiking activity from many single neurons isolated from the 96-channel multielectrode array using a Cerebus Neural Signal Processor (Blackrock Microsystems). A block of 32 channels could be recorded simultaneously over the course of a session. The raw signal was bandpass filtered (0.3 Hz to 7.5 kHz) and digitized (16 bit) at 30,000 samples/s. For each channel, spikes were detected every time the digitally high-pass filtered (250 Hz/4-pole) voltage trace crossed a threshold equivalent to approximately four times the root mean square of the noise amplitude. The extracted spikes and associated waveforms were sorted offline using both manual and semi-automatic techniques (Offline sorter, Plexon Inc.). Monkey “F” completed 27 recording sessions with a mean (STD) of 47.89 (6.67) simultaneously recorded neurons. Monkey “JL” completed 28 recording sessions with a mean of 51.89 (3.67) simultaneously recorded neurons.

### Drug administration

The pharmacokinetics and bioavailability of MPH in humans and monkeys have been described in detail previously and guided our selection of dose range and administration schedule (Wargin et al., 1983; Doerge et al., 2000). The peak serum concentration of MPH is attained ∼60 min after oral administration in monkeys, with a half-life of 1.79 h. Therapeutic serum concentration is already obtained 30 min after oral administration in monkeys, which agrees with the delay to clinical onset in children ranging from 20 to 60 min, and lasting 3–5 h (Kimko et al., 1999; Doerge et al., 2000). Moreover, clinical evidence shows that the optimal dose of MPH for children with ADHD is patient specific, with best dosages ranging from 5 to 60 mg/d (Subcommittee on Attention-Deficit/Hyperactivity Disorder et al., 2011). Therefore, we expect that each monkey in this experiment might best respond to a different dose. Based on the above information and on a previous study in monkeys demonstrating MPH-dependent behavioral improvements with best doses ranging from 0.1 to 1.2 mg/kg (Gamo et al., 2010), we tried a range of drug doses to find a dose that best improved performance at the attentional task in each animal. We orally administered 2.5, 5, 6.25, 7.5, or 10 mg short-acting tablets of MPH (Ritalin, Novartis) to each animal, which corresponds to a weight-based dosing of 0.43, 0.86, 1.08, 1.29, or 1.72 mg/kg for monkey “F,” and 0.33, 0.67, 0.83, 1.00, or 1.33 mg/kg for monkey “JL.” We orally administered MPH or placebo to the monkeys 30 min before the beginning of a behavioral testing session. In treatment sessions, we diluted MPH into 5 ml of concentrated fruit juice vehicle and gave the juice to the monkey orally using a syringe. Because our monkeys were under a water control schedule between sessions, they always drank the entire content of the syringe immediately when offered. A given dose was given to our subjects for three consecutive recording sessions (one session per day) to control for normal day-to-day variation in behavioral performance. A block of three treatment sessions was preceded and followed by a block of two placebo sessions (one session per day). This bilateral flanking of treatment sessions with placebo sessions (Pb-Pb-MPH-MPH-MPH-Pb-Pb) allowed a more robust MPH-Placebo comparison by controlling for low-frequency confounds on performance, such as task learning or overall motivation to perform the task. In placebo sessions the experimental procedures were identical except for the fact that the concentrated fruit juice administered before the session did not contain MPH. Out of 27 sessions, monkey “F” completed 15 sessions with MPH and 12 with placebo, whereas monkey “JL” completed 16 sessions with MPH and 12 sessions with placebo out of 28 sessions in total.

### Data analysis

All data analyses were conducted using custom scripts written in MATLAB (MathWorks Inc.), and standard operations in Excel (Microsoft Inc.) and SPSS (IBM Inc.). Throughout the analyses, the data from each monkey were analyzed separately and was not averaged across monkeys. This allowed detecting potential interindividual differences in drug response, both at a behavioral and neurophysiological level. This also provided a mean to look for patterns of drug dose-responses that are consistent across monkeys, providing an additional protection against false positive results through direct replication in a second animal. For each monkey individually, the behavioral data of sessions with the same MPH dose (a block of three consecutive sessions with a given dose) was pooled to compute the performance statistics (hit rate). The same was done for placebo sessions flanking each block of treatment sessions, such that the performance under each dose of MPH could be directly compared with the performance during flanking control sessions. Behavioral performance was compared between MPH and placebo sessions by comparing hit rates [Hits/(Hits + Misses)] for a block of drug sessions and matched placebo sessions using Pearson’s χ^2^ tests (χ^2^) for differences in proportions.

The tuning, or “selectivity,” of single neuron responses for the cue position, the allocation of selective attention, and the saccade goal were computed during the three corresponding task epochs (cue, attention, and saccade) during “target only” trials. We used a Kruskal–Wallis one-way ANOVA on the neuronal firing rate across the four possible target locations to determine the preferred and anti-preferred visual quadrant, and to define whether each neuron is considered “selective” or “non-selective” during each task epoch (cue, attention, and saccade), based on the significance of the test evaluated at *p* < 0.01.

Spike density functions (SDFs) for each neuron were obtained by convolving the spike train of each trial with a Gaussian kernel with a SD of 30 ms. Trial-averaged SDF were obtained by averaging the resulting time series across all trials of the same task condition, each one of the four target positions being considered one condition. Normalized population responses for each condition were obtained by z-scoring the trial-averaged SDF of each individual neuron and then pooling across neurons.

From the trial-averaged SDF of each neuron, 19 single neuron response metrics were computed across the three task epochs and compared across treatment conditions to detect potential effects of MPH on single neuron activity. For each epoch, these metrics were computed only on selective neurons for the corresponding epoch.

#### Visual epoch

(1) The “baseline firing rate” was computed from an interval of 200 ms before cue onset by averaging the neuronal activity in this time window. (2) The “peak cue-elicited response” was computed by finding the trial-averaged peak response in the preferred quadrant from a time window of 200 ms after cue onset. (3) The “latency of cue-elicited response” was calculated by measuring the time interval between cue onset and peak response in the preferred quadrant. (4) The “Fano factor of peak visual response” is the coefficient of variation, or the mean-normalized SD, of the sample of peak cue-elicited responses over trials. (5) The “attentional modulation of visual response” is the ratio between the average peak visual response when an attended target stimulus appears within the preferred quadrant of the neuron, and the average peak visual response when a non-attended distractor appears within the preferred quadrant of the neuron. (6) The “peak distractor-elicited response” is the average peak visual response elicited by a distractor appearing within the preferred quadrant of the neuron. (7) The “latency of distractor response” is the time interval between the distractor stimuli onset and the peak distractor-elicited visual response. (8) The “Fano factor of peak response” is the coefficient of variation of all peak cue-elicited responses when the peak timing is determined on a single-trial basis rather than on a trial average peak response. (9) “Receptive field (RF) modulation of visual response” is computed using the ratio of the peak response when the cue appears inside the preferred quadrant, and the peak response when the cue appears in the anti-preferred quadrant. (10) The “peak response to anti-preferred” is the peak cue-elicited response when the cue appears in the anti-preferred quadrant. (11) The “latency of the anti-preferred response” is the time interval between the onset of the cue and the peak cue-elicited response when the cue appears in the anti-preferred quadrant.

#### Attention epoch

(12) The “sustained response when attention is in the RF” is the average response over a time window of 400 ms in the middle between cue offset and saccade onset when the monkey is allocating covert visual attention inside the RF (i.e., preferred quadrant) of the neuron. (13) The “sustained response when attention outside the RF” is the same as 12, but when covert visual attention is allocated outside the RF (i.e., in the anti-preferred quadrant). (14) The “attentional modulation of sustained response” is the ratio between 12 and 13, i.e., the ratio between the response when attention is allocated inside versus outside the RF of the neuron. (15) The “Fano factor of sustained response” is the Fano factor of the sustained response described in 12, i.e., when covert attention is allocated inside the RF of the neuron.

#### Saccade epoch

(16) The “peak saccadic response” is the peak response aligned to saccade onset when the saccade is made toward the preferred quadrant of the neuron. (17) The “peak anti-preferred saccade” is the peak response aligned to saccade onset when the saccade is made toward to anti-preferred quadrant of the neuron. (18) The “saccadic response modulation” is the ratio between 16 and 17, i.e., when saccades are made toward versus opposite to the preferred quadrant. (19) The “Fano factor of the saccadic response” is the Fano factor of the peak saccadic response when directed toward the preferred quadrant.

A “noise correlation” is the trial-to-trial spike count correlation between two neurons’ simultaneous activity (Cohen and Kohn, 2011). This correlation is called “noise” because it is computed using the variance over trials of the same stimulus condition, therefore modeling the error, or noise, around the mean response for a given stimulus condition. This is in contrast with the “signal correlation” which is computed across trials of different stimulus conditions. In this latter case, the correlation is computed between two neurons’ average response across all stimulus conditions, and can be thought of as a measure of tuning similarity. Noise correlations were computed in our sample between all possible pairs of simultaneously recorded neurons using the Pearson’s correlation coefficient *r*. A series of noise correlations were computed as a function of two experimental factors. First, noise correlations were computed for each of the four task epochs (baseline fixation, visual epoch, attention epoch, and saccade epoch). Second, noise correlations were computed either on all recorded neurons, or only on selective neurons for the corresponding task epoch. Noise correlations computed over sessions with the same drug dose were pooled together in a single distribution. From these distributions, the median noise correlation coefficient was reported for the sub-population of positive coefficient, and for the sub-sample of negative coefficient. Keeping positive and negative noise correlations separate is important since these two have different physiologic interpretations (i.e., shared or direct excitatory input in the case of positive, and shared input of opposite valence or direct inhibitory input in the case of negative noise correlations). To ease visualization of potential effects of the drug on noise correlations, the percentage change from placebo was calculated and reported for all series of noise correlations. Finally, we describe the “noise correlation structure” in our neuronal populations, which is the relationship between noise correlations and tuning similarity between neurons. Neurons that have similar tuning are expected to share more common inputs or make more direct connections, thus increasing their noise correlation (Cohen and Kohn, 2011). We computed the relationship between signal correlation and noise correlation for placebo sessions and all different drug doses to detect potential MPH-dependent differences in the noise correlation structure.

To assess the quality of the attentional filtering in area 8A’s neuronal population activity, we used a support vector machine (SVM) linear decoder to which we fed the simultaneously recorded firing rates of the each recorded neuronal ensemble (Chang and Lin, 2011). A neuronal ensemble is a population of simultaneously active neurons involved in the same neuronal computation (Hebb, 1949; Nicolelis et al., 1997). Using this method, it was recently possible to decode the focus of covert attention using the instantaneous activity of ensembles of recorded neurons in the macaque caudal LPFC (Tremblay et al., 2015a,b). Here, we used this method to quantify the amount of attentional information that can be extracted from the neuronal ensemble activity (Quian Quiroga and Panzeri, 2009), and to compare it across drug treatments to detect potential MPH-related effects on the attentional filtering implemented by prefrontal neuronal ensembles. The algorithm’s decoding accuracy of the focus of attention was used as a proxy for the coding efficiency of the neuronal ensembles and was computed using a cross-validation procedure where 4/5 of trials are used for training the algorithm and the remaining 1/5 of trials are used to test the decoder’s predictions, iteratively (for detailed method, see Tremblay et al., 2015b). The statistical significance of the decoding accuracies for each task epochs and each drug dose were tested against a control condition where the labels of each trials were iteratively permuted randomly during the supervised training phase of the machine learning algorithm (*N* = 1000 permutations). To better visualize the potential MPH-dependent effects on neuronal ensemble coding of attention in area 8A, a percentage change in decoding accuracy from placebo was also computed. This ratio was computed using flanking placebo sessions recorded immediately before and immediately after each set of treatment sessions using a given drug dose. This was done to control for low-frequency variations in neuronal ensembles’ composition over time.

### Experimental design and statistical analysis

Across the manuscript, data from both monkeys was never combined or averaged. This allows a direct comparison between the results of the two monkeys and to assess the replicability of observations across subjects. It also avoids the problem analyzing nested data, which require specific statistical corrections (Aarts et al., 2014). Only trends that replicated across both monkeys were considered true effects. Bonferroni corrections for multiple comparisons have been applied where necessary. No corrections have been applied to results that were non-significant even before statistical correction (these uncorrected results would remain null after correction). Effect sizes have been reported where statistical power is too high for *p* values to be meaningful (e.g., correlation analyses). If this concept is not clear to the reader, please consult the following resource (Wasserstein and Lazar, 2016).

## Results

### Behavioral performance

We compared the monkeys’ behavioral performance at the task during MPH sessions with performance during matched placebo sessions. Overall, both monkeys performed well above chance (chance = 25%, four options) during placebo sessions (Fig. 1*B*). In monkey “F,” MPH slightly enhanced performance when administered at 0.86 mg/kg (*p* < 0.05, χ^2^ test). A lower dose had no effect on performance, and higher doses decreased performance relative to placebo (*p* < 0.05, χ^2^ test; Fig. 1*C*). In monkey “JL,” the best dose was 0.67 mg/kg (*p* < 0.001, χ^2^ test; Fig. 1*C*). A lower dose had no effect on performance and higher doses had either null or smaller positive effects (*p* < 0.01, χ^2^ test; Fig. 1*D*). These variable, weak behavioral results are comparable to those reported by previous studies in monkeys where MPH was reported to have a weak, subject-specific, but statistically significant effect on cognitive performance (Prendergast et al., 1998; Bain et al., 2003; Gamo et al., 2010; Rajala et al., 2012). We did not find MPH to have specific effects on one type of error, whether impulsivity or distractibility errors (Fig. 1*E*,*F*) in either monkey. Both types of errors were affected by the drug at various levels (*p* < 0.05, χ^2^ test).

### Single neuron analyses

**Figure 3.**
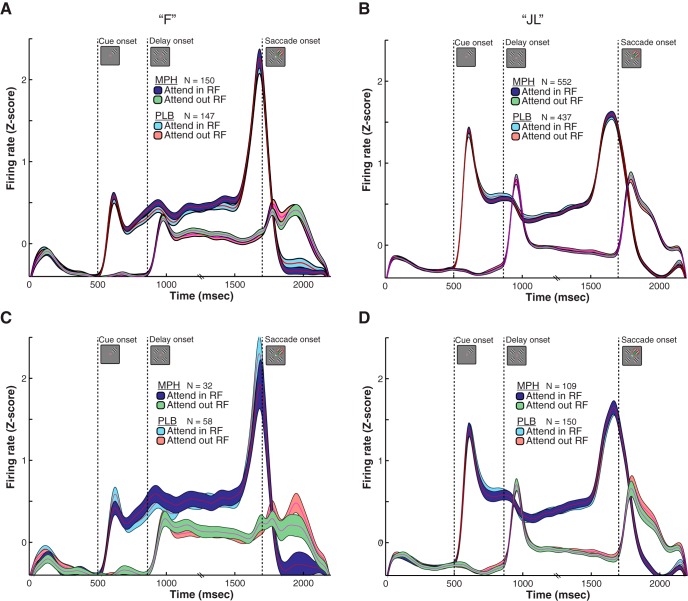
Qualitative effects of MPH on the average single neuron response. ***A***, Attentional modulation of single neuron activity averaged over the entire sample of tuned cells and trials (sample size reported with *N* in figure). The trial-averaged SDFs are displayed separately for MPH and placebo (PLB) sessions. The light blue and red SDFs depict the average single neuron response on trials where attention is allocated inside, or opposite to the neuron’s preferred location (i.e., RF), respectively. The abscissa represents the time from trial onset and the ordinate the population neuronal firing rate (z-scored). Shaded areas represent SEM. The average responses during all MPH sessions are overlaid on top of the average response during placebo sessions to illustrate the near-perfect overlap in single neuron responses across treatment conditions. ***B***, Same as in ***A*** but for monkey “JL.” ***C***, ***D***, Same as ***A***, ***B*** but only including the MPH sessions showing the best behavioral improvement due to treatment (best-dose analysis; 0.86 mg/kg for monkey “F,” 0.67 mg/kg for monkey “JL”). The same absence of difference in this best-dose analysis is demonstrated by the overlap of the MPH and PLB curves in ***C***, ***D***.

First, we analyzed the effects of all doses of MPH on the responses of single neurons. To qualitatively detect any main effect of MPH (pooled across all doses) on the average response profile of the sampled neuronal population, we overlapped the population-averaged SDF for placebo sessions with the population-averaged SDF during all MPH sessions (Fig. 3*A*,*B*). This sample of neurons included only neurons that were attention-selective (i.e., that are modulated by visual attention) allowing to visualize the attentional modulation and potential effects of MPH on this modulation. To illustrate the attentional modulation in this sample of neurons, we computed the average response when the attended stimulus/target was inside the RF (attend in RF in Fig. 3), and when the stimulus was outside the RF (attend out RF in Fig. 3), as is routinely done in basic attention research. The two response profiles greatly differ during the delay epoch depending on the focus of attention (in or outside the RF) despite identical stimulus presentation and motor state during this epoch (all four grating stimuli were present on the screen while the monkey was fixating on the center dot). This modulation is the trademark of visual selective attention at the single neuron level, although it is difficult to disentangle the contribution of saccade planning from visual attention using visual-saccadic paradigms such as ours (Reynolds and Chelazzi, 2004). MPH, however, does not seem to modify the average attentional response profile, as shown by the near-perfect overlap between the SDF of similar conditions during placebo and MPH sessions in both monkeys (Fig. 3*A*,*B*). The same is true when placebo sessions are contrasted only to MPH sessions showing a positive behavioral effect of the drug (Fig. 3*C*,*D*).

**Figure 4.**
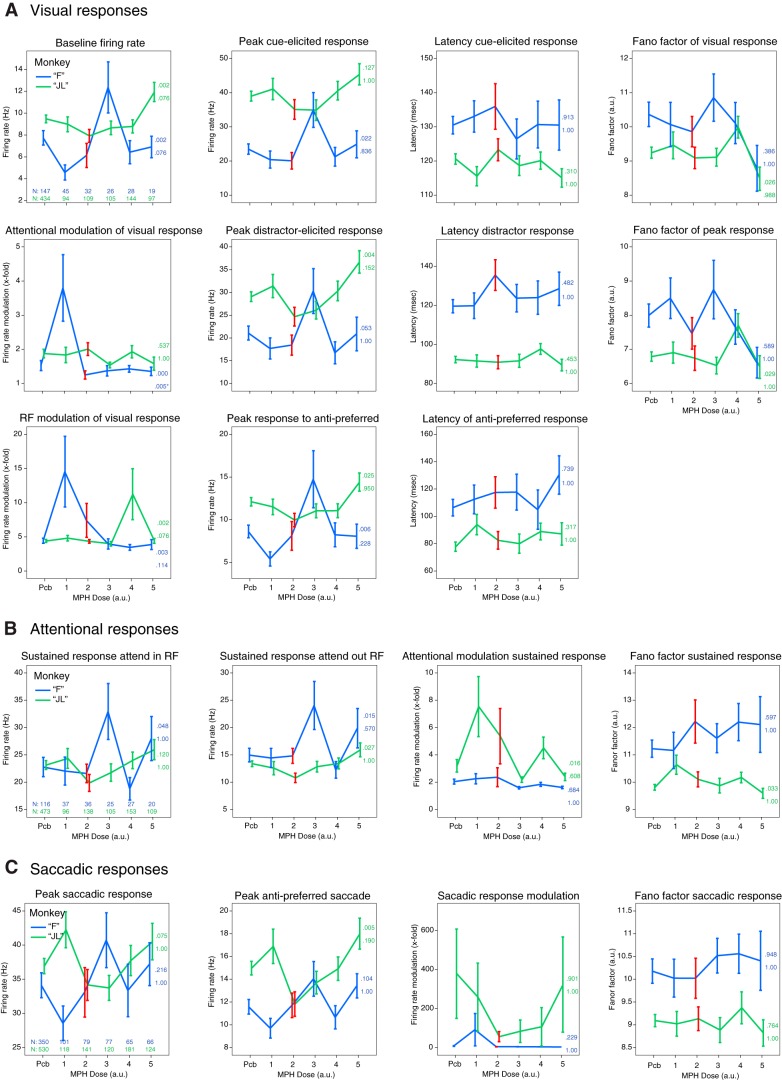
Effects of MPH on 19 single neuron response metrics. ***A***, Visual response metrics for visually-selective neurons as a function of drug dose. Refer to Materials and Methods for the meaning of each metric. The *x*-axis depicts MPH drug dose using arbitrary units (a.u.), from the smallest dose to the biggest for each monkey. These are 0.43, 0.86, 1.08, 1.29, or 1.72 mg/kg for monkey “F,” and 0.33, 0.67, 0.83, 1.00, or 1.33 mg/kg for monkey “JL.” The *y*-axis is relative to the particular metric being plotted. Blue and green lines are for monkey “F” and monkey “JL,” respectively. The top-leftmost subplot includes the size of single neurons samples included for the computation of all the visual metrics. The red error bars correspond to the best-dose of MPH according to behavioral performance. The colored numbers to the right of each line represent the *p* values for the ANOVA test ran for each metric, uncorrected (top), and corrected for multiple comparisons (bottom). ***B***, Same as in ***A*** but for the attentional response metrics of attention-selective neurons. ***C***, Same as in ***A*** but for the saccadic response metrics of saccade-selective neurons. All error bars represent SEM.

To quantify these qualitative observations, we further characterized the response profile of each recorded single neuron (*N* = 2811, including multiunit clusters) using 19 response metrics for all doses independently (see Materials and Methods for details on metrics). In Figure 4, we present the results for all 19 response metrics as a function of monkey, task epoch, and drug dose. Each point represents the average metric across all neurons that met the criteria for the specific analysis (e.g., modulation by attention; see Materials and Methods). We looked for any dose-response effect of the drug, whereby increasing doses produce a more profound deviation (negative or positive) from placebo. The dose-response curve did not need to be linear (e.g., U-shaped curves were considered). As a protection from spurious findings, the dose-response curve had to be replicated in both monkeys. However, the curve could be shifted horizontally across subjects to account for subject-specific best dose responses. Across all 19 metrics, we did not find a single metric that satisfied both criteria above. We computed one-way ANOVAs with “dose” as the factor on each metric to test for statistically significant effects of MPH. We defined a significant effect as an effect that passed a Bonferroni correction for multiple comparisons, and that was found in both animals. All metrics failed to cross a statistical threshold of *p* < 0.05 following Bonferroni correction for multiple comparisons (38 tests), except one: “attentional modulation of visual response” (Fig. 4*A*), where 2.5 mg in monkey “F” produced a significantly higher score (*F*_(5,290)_ = 5.2, *p* < 0.001) than placebo. Since this finding was not replicated in the other monkey at any other dose and was not embedded in a larger dose-dependent trend, it was considered a spurious finding. Overall, these quantitative analyses corroborate what was observed qualitatively in Figure 3, that is, that MPH does not seem to reliably modify single neuron responses.

### Noise correlations

**Figure 5.**
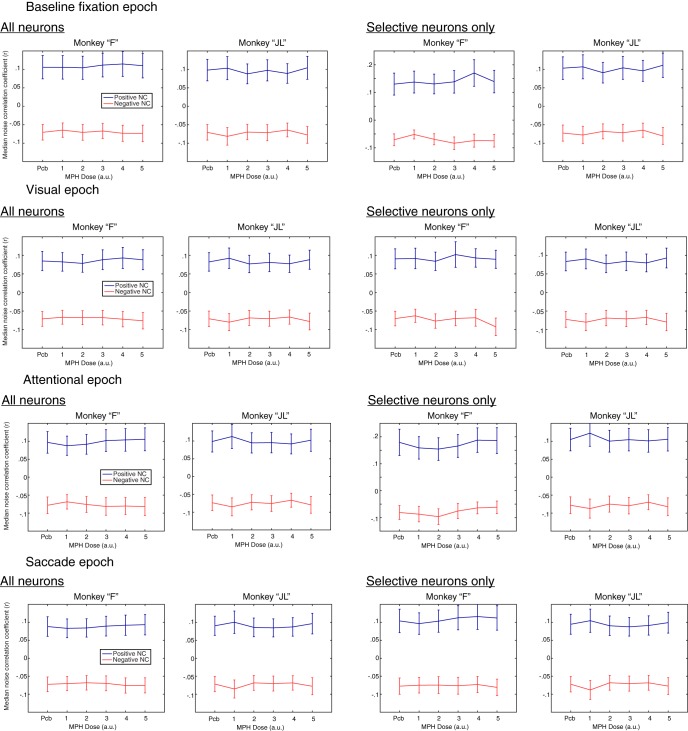
Effects of MPH on noise correlations. Each line illustrates the median noise correlation coefficient separately for positive and negative noise correlations (blue and red, respectively) as a function of drug dose (*x*-axis). The *x*-axis uses arbitrary units, from the smallest dose of MPH to the biggest for each monkey. These are 0.43, 0.86, 1.08, 1.29, or 1.72 mg/kg for monkey “F,” and 0.33, 0.67, 0.83, 1.00, or 1.33 mg/kg for monkey “JL.” The left column presents results from analyses including all recorded neurons, independent of their selectivity. The right column includes only neurons that were selective (i.e., tuned) for the corresponding epoch. Since no tuning can be measured during the baseline fixation epoch, visual tuning was used as a replacement in this analysis. Each column presents results for each monkey independently.

A possible explanation for the cognitive-enhancing effects of MPH is that the drug decreases correlated noise between neurons (noise correlations). To test this hypothesis, we analyzed the correlated activity from an average of 48 simultaneously recorded neurons in monkey “F” and 52 neurons in monkey “JL” across all 55 recording sessions. We computed the noise correlations between all possible pairs of simultaneously recorded units from different electrodes within the multielectrode array (excluding neurons recorded from the same electrode). We computed those correlation coefficients (Pearson’s R) for all four task epochs separately (baseline fixation, visual, attention, and saccade; Fig. 5). Whereas this first analysis included all neurons irrespective of their tuning, we also replicated this analysis for the subgroup of task-selective neurons specific to each epoch (e.g., attention-selective neurons during the attention epoch).

As for the single neuron analysis above, we searched for results that would (1) show a dose-response trend, and (2) replicate across the two monkeys, with some flexibility on the horizontal shift across doses. Again, the results were inconclusive for all epochs, and neuronal sample (all neurons or only those selective; Fig. 5). To substantiate this qualitative assessment, we ran one-way ANOVAs with dose as the factor for each epoch, neuronal group, correlation sign (positive or negative), and monkey. All ANOVAs produced *p* values < 1.0 × 10^−50^. Obviously, such a statistical significance is a consequence of the very large sample size of correlations rather than the size of the effect of MPH (>5000 correlations for each test; one noise correlation per possible pair of neurons). With such a large sample size comes an inflated statistical power. In this context, statisticians advice that effects sizes need to be interpreted to assess the importance of the effect instead of only relying solely on *p* values (Cohen, 1992; Lin et al., 2013; Wasserstein and Lazar, 2016). When computing effect sizes for each ANOVA, we find that no model provides >2% of explained variance (η^2^ < 0.02). In other words, MPH doses account for <2% of the variability observed in noise correlations across sessions.

**Figure 6.**
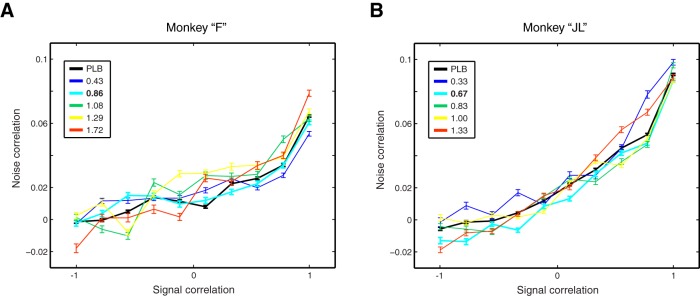
Effects of MPH on noise correlation structure. ***A***, Relationship between the signal correlation (i.e., tuning similarity) and noise correlation between every possible pairs of simultaneously recorded neurons, presented for each drug dose (colored lines). As expected, the more similar is the tuning between two neurons, the more noise they share through common inputs. ***B***, Same as in ***A***, although for monkey “JL.” Best dose of MPH based on behavioral performance is in bold in the legend. Error bars represent SEM.

Noise correlations between a pair of neurons can be modulated by the tuning similarity of those two neurons. The function that links these two variables (noise correlation and tuning similarity) is considered as the noise correlation “structure” of the recorded neuronal population. This structure could be modulated by MPH as a mechanism of action of the drug. We plotted this relationship for each drug dose for each monkey separately (Fig. 6). We found that when neurons differ the most in tuning (signal correlation close to –1), the average noise correlations are close to zero or slightly negative. Moreover, the average noise correlation coefficient increases proportionally with the tuning similarity of pairs of neurons, which is to be expected from neurons sharing more common inputs. To investigate the effects of MPH on this correlation structure for both subjects, we ran one-way ANOVAs with dose as the factor for 10 signal correlation “groups” (each data point on the *x*-axis of Fig. 6 is for one group). These groups simply pool similar signal correlations together according to the following intervals: [–1 to –0.8], [–0.8 to –0.6], etc. Similarly to the above noise correlation analyses, each dose group contained >10,000 correlations, inflating statistical power beyond the point where *p* values are interpretable. As expected, all *p* values computed with the ANOVAs converged to zero (all *p* < 1.0 × 10^−50^). When evaluating the effect sizes (η^2^) in addition to *p* values, we found that no group comparison yielded >1% of explained variance (all η^2^ < 0.01). In other words, MPH doses account for <1% of the variability observed in noise correlations across the spectrum of tuning similarity.

### Ensemble decoding

MPH could improve the neuronal encoding of spatial attention by increasing the reliability of neuronal ensembles’ activity in the LPFC rather than modifying single neuron or pairwise metrics. It is also possible that very small changes in the correlation structure of these ensemble can have an impact on the quality of the neuronal representation (Shadlen et al., 1996; Moreno-Bote et al., 2014; Kanitscheider et al., 2015). To investigate this possibility, we used a SVM linear decoder to extract the allocation of attention, visual stimulation, and saccadic eye movements from the responses of large ensembles of neurons recorded simultaneously (see Material and Methods). Using this methodology, we previously found that it was possible to decode the focus of spatial attention from ensembles of prefrontal neurons and that this representation was sensitive to distractors and predictive of upcoming attentional mistakes (Tremblay et al., 2015a,b).

**Figure 7.**
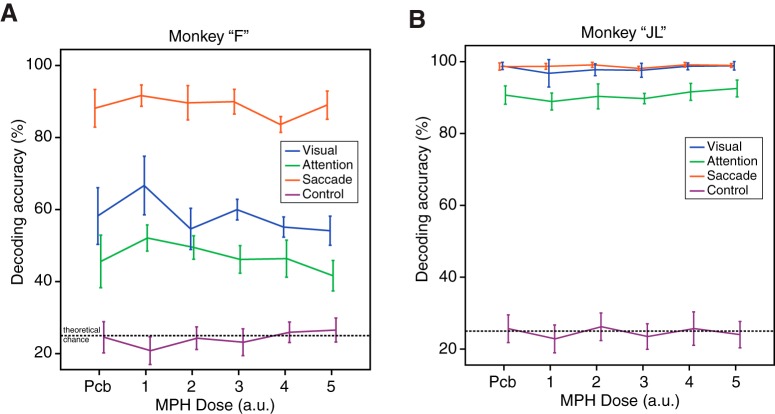
Effects of MPH on neuronal ensemble decoding of task-related information. ***A***, Decoding accuracy of a SVM algorithm extracting single-trial information about the visual, attentional, and saccadic representations in the neuronal ensemble activity of simultaneously recorded neurons. Decoding accuracy, used as a proxy for neuronal coding accuracy, is presented as a function of drug dose (*x*-axis), as in preceding figures. The purple line represents the achieved chance performance using permutation testing and overlaps roughly with the theoretical chance performance of 25%. ***B***, Same as ***A*** but for monkey “JL.” Error bars represent SEM.

When applying those machine learning techniques to the current dataset, we found that visual stimulation, spatial attention, and saccadic eye movements could be decoded from the single-trial information contained in the instantaneous firing of neuronal ensembles. The accuracies varied from 40% to 100%, all above the change decoding accuracy of 25%. When looking at the effects of MPH on the coding accuracy of attention, visual stimulus location and saccade endpoint, we found inconsistent effects (Fig. 7). Qualitatively, we observed neither a dose-response effect of MPH administration, nor any effects that replicated in both monkeys. Quantitative analysis of the effect of MPH on the decoding accuracy of visual, attentional, and saccadic information revealed no statistical differences using one-way ANOVAs with dose as the factor (monkey “F”; visual: *F*_(5,21)_ = 1.5, *p* = 0.23, attention: *F*_(5,21)_ = 1.3, *p* = 0.32, saccade: *F*_(5,21)_ = 1.1, *p* = 0.41; monkey “JL”; visual: *F*_(5,22)_ = 0.86, *p* = 0.52, attention: *F*_(5,22)_ = 0.92, *p* = 0.49, saccade: *F*_(5,22)_ = 0.83, *p* = 0.54), even before correction for multiple comparisons.

## Discussion

Contrary to our hypothesis, our results support that oral administration of MPH does not produce detectable effects on the neurophysiology of the caudal PFC in monkeys, a brain region otherwise known for its critical role in attention as demonstrated by microstimulation, pharmacological, and optogenetic studies in primates (Dias and Segraves, 1999; Moore and Fallah, 2004; Noudoost and Moore, 2011; Schafer and Moore, 2011; Acker et al., 2016). Since the vast body of literature from rodent research all point to the PFC as the main site of action of MPH (Berridge and Arnsten, 2015; Spencer et al., 2015), we are surprised that no systematic effects were detected at the single, pairwise, or neuronal ensemble level in the current study in nonhuman primates. In search for effects of MPH on single neuron activity, we have performed all mainstream neurophysiological analyses common to basic attention research in primates, and found no consistent effect. Noteworthy, this absence of effect is observed even at doses that increase the monkeys’ behavioral performance. To our knowledge, this is the largest neurophysiological investigation of MPH to be performed in nonhuman primates with >2800 neuronal recordings [the only other study by Gamo et al. (2010) recorded from 17 neurons). We believe these negative results deserve to be shared with the community and new directions of research into the mechanism of action of MPH ought to be discussed.

### Neuroanatomical considerations

This series of negative neurophysiological findings does not support the hypothesis that MPH would affect both single neuron and correlated noise activity patterns in the primate dorsolateral PFC. Our choice of brain region for neuronal recordings was based on solid evidence from neurophysiological studies in nonhuman primates demonstrating that this brain area and the adjacent frontal eye fields (FEFs) are areas robustly associated with visual selective attention (Noudoost and Moore, 2011; Schafer and Moore, 2011; Squire et al., 2013; Clark et al., 2015; Tremblay et al., 2015b). In parallel, neuropharmacology and neurophysiology research in rodents have identified the PFC as the primary target of MPH. Studies in rodents have provided compelling evidence that MPH affects preferentially the neurochemistry and neurophysiology of the PFC at low doses that improve cognitive performance, with little effects outside the PFC (Berridge et al., 2006; Devilbiss and Berridge, 2008; Spencer et al., 2012, 2015; Berridge and Arnsten, 2015). We do not believe our study challenges the results from those rodent studies nor questions the results from basic attention research in nonhuman primates. We propose, however, that our negative findings might arise from neuroanatomical considerations when translating pre-clinical results from the rodent brain to the primate brain.

The PFC is a vast landscape with several sub-regions both in the macaque monkey brain and in the human brain. These distinct anatomic areas within the PFC are differentiated both by their cytoarchitecture (Barbas and Pandya, 1989; Preuss and Goldman-Rakic, 1991; Petrides and Pandya, 1994), their corticocortical connectivity and sub-cortical projection pattern (Petrides and Pandya, 1999, 2006), and their specific behavioral consequences following lesions, which allow establishing double-dissociations within that landscape (Petrides, 2005; Simmons et al., 2010; Rudebeck and Murray, 2011; Rudebeck et al., 2013). The target area in the current study is only one of many subdivisions in the primate PFC. Therefore, our results do not rule out the possibility that if we had placed our multielectrode arrays in a different area of the PFC (for example, area 9/46) we would have been able to capture neurophysiological effects of MPH similar to the ones previously reported in the rodent literature. However, we are doubtful of such a proposition because area 9, 46, and 9/46 of the primate dorsolateral PFC are more robustly associated with working memory and monitoring within working memory rather than attentional processes (Goldman-Rakic, 1995; Curtis and D'Esposito, 2004; Petrides, 2005; Leavitt et al., 2017a).

An important question to answer is whether there is a homolog of the caudal dorsolateral PFC in the rodent brain. This question might be particularly difficult to answer since there exists no brain map that describes neuroanatomical homologies between rodent and primate PFCs. Many career neuroanatomists would even question the presence of a homolog of the primate PFC in the rodent brain since the rodent frontal cortex lacks the granularity (i.e., presence of stellate cells in Layer IV) that is the hallmark of the PFC in primates, including humans (Petrides and Pandya, 1994; Palomero-Gallagher and Zilles, 2004). This absence of demonstrated homology poses a serious problem when researchers attempt to bridge the two separate worlds of primate and rodent PFC research. Our study is no exception.

It may be more appropriate to interpret our results within the framework of human neuroimaging studies that provide indirect measures of MPH activity (e.g., using positron emission tomography; PET). As opposed to results from rodent research, findings from this literature propose several targets within and outside the PFC where MPH can elicit its effects. Indeed, systemic administration of MPH in humans leads to changes in regional cerebral blood flow (rCBF) both inside and outside the PFC. Areas including the striatum, the supplementary motor area and the posterior parietal cortex are significantly modulated in healthy human subjects undergoing PET imaging following administration of clinically relevant doses of MPH (Mehta et al., 2000; Volkow et al., 2005). In parallel, pharmacological studies analyzing the occupancies by MPH of the dopamine and norepinephrine transporters (both primary targets of MPH) find a high level of binding in several cortical and sub-cortical areas outside the PFC, such as the thalamus and striatum (Volkow et al., 1998; Rosa-Neto et al., 2005; Hannestad et al., 2010). Similar results have been reported using functional magnetic resonance imaging (Peterson et al., 2009; Tomasi et al., 2011) and electroencephalography (Dockree et al., 2017) in humans, suggesting that the MPH has widespread effects in several brain regions. Given those potential targets of MPH identified from human research, one might ask why we chose to record from the PFC in our monkeys. The answer is twofold: (1) the multielectrode arrays we use to record from many neurons simultaneously and measure noise correlations cannot be implanted in sub-cortical structure or deep sulci, and (2) the PFC remains the only brain area that is modulated by MPH both in human and rodent research, making it the most logical first target for our investigation.

Importantly, a recent microdialysis study in monkeys found no effects of cognitive-enhancing doses of MPH on dopamine release in the monkey PFC in conjunction with an increase of dopamine release in the striatum (Kodama et al., 2017). Dopamine release modulation is one of the mechanisms through which MPH is thought to exert its effects on neurophysiology (Arnsten and Dudley, 2005). Our findings, which show an absence of effect of MPH on prefrontal neurophysiology, at least partly agree with the negative findings reported by Kodama et al., on prefrontal neurochemistry. Taken together, these two converging sets of observations ask for further investigations into the effects of MPH on the primate brain. It is worth noting, however, that the study by Kodama et al. (2017) focused on a slightly more anterior area of the monkey LPFC encompassing the principalis sulcus (area 8A, 9/46, and 46).

### Study limitations

Our study includes some limitations worth discussing. First, as discussed above, the use of multielectrode arrays is a powerful way to measure the activity of large population of neurons on a realistic timescale, but it only provides a field-of-view of a few millimeters squared. Our conclusions therefore can only be applied to the most caudal aspect of the PFC in monkeys. Second, there is a significant amount of variation from session to session in the behavioral performance of the monkeys. On the statistical level, this variability makes it difficult to detect behavioral effects of MPH since the small effects of the drug could be masked by normal, random day-to-day variations in behavioral performance. This normal variation in the performance of monkeys is not a problem specific to the current study, but is rather characteristic of the work with those highly intelligent animals who can be motivated, or distracted, by many difficult to control factors.

On this note, Soto et al. (2013) provide an important warning regarding potential statistical flaws when using a best-dose analysis to identify the optimal dose of a cognitive-enhancing drug on a subject-by-subject basis. We took precautions to prevent such flaws by retesting every dose three times in each animal. We are confident, nonetheless, that our drug administration procedure was reliable given that the water-controlled monkeys always consumed the juice containing the drug entirely and immediately when given. Therefore, regardless of the results of the behavioral analyses on task performance, we are confident that various doses of MPH, including clinically relevant doses, were administered systematically to our subjects. In other words, the neurophysiological study of MPH could be performed even without performance of a behavioral task and yield important insights on its mechanism of action. This is the reason why we also analyzed the neurophysiology of sessions where no behavioral effects of the drug were observed.

On the neurophysiological level, one could argue that it would have been preferable to administer a placebo followed by the drug within each recording session to provide a within-session comparison of placebo and MPH on both behavioral and neuronal levels. Although we agree that this is a possibility, we want to point out that this within-session method permits only comparing MPH to placebo when MPH came second after placebo within a session, and not when MPH came before placebo, which is a major methodological problem. The second order of administration (1st: MPH, 2nd: PLB) is impracticable due to the long half-life of MPH (Volkow et al., 1995) which would have contaminated the placebo condition if recorded in the same day. Counterbalancing the order of administration of drug and placebo in a within-subject design is crucial. For example, the performance of monkeys usually decrease within a session as their motivation wears down when they become gradually satiated with the reward. The within-session design would not have allowed to control for those major confounding variables, whereas our between-session design did. What our design failed to achieve is a better statistical power associated with paired statistical tests when comparing a neuron to itself under various drug conditions (using a paired *t* test for example). In our design, the large inter-neuronal variability could only be counter-balanced by larger neuronal sample sizes. It could be, also, that the effects of MPH are noticeable only in certain neuronal types. This is not something our analyses could have detected, unfortunately.

In conclusion, we propose that future research investigates the neurophysiological effects of MPH in the monkey brain by exploring other areas of the PFC, as well as other areas of the attentional network, such as the striatum and the posterior parietal lobe (e.g., the lateral intraparietal area). We do advise investigators, however, that these future neurophysiological studies should be conducted using multielectrode recording technologies. From what we currently know of basic attention mechanisms in the brain, correlated noise between neurons appears to be the main modulator of attentional processing at the cellular level. This correlated noise can only be detected by recording from many neurons simultaneously, and might be the primary target of MPH. We also propose that a dialogue should be maintained between rodent and monkey researchers to find better ways to translate neurophysiology results across animal models and build bridges between those scientific communities.

## Synthesis

Reviewing Editor: Tatiana Pasternak, University of Rochester Decisions are customarily a result of the Reviewing Editor and the peer reviewers coming together and discussing their recommendations until a consensus is reached. When revisions are invited, a fact-based synthesis statement explaining their decision and outlining what is needed to prepare a revision will be listed below. The following reviewer(s) agreed to reveal their identity: Bruno Averbeck.

The paper was reviewed by two experts. Initially, there was a disagreement among the two reviewers. While Reviewer 1 raised relatively few concerns, Reviewer 2 had a number of more serious reservations. In the subsequent discussion, the agreement was reached concerning the need for compelling justification for selecting the region in the PFC to examine drug effects. I am going to ask you to revise the manuscript and address this and several other issues raised by the reviewers, before the paper can be considered for publication in eNeuro. Below is the list of problems that will need to be addressed in the revision.

1. Please provide arguments in support of the choice of area 8A to study the effects of MPH and of the expectation that neurons in area 8A would be affected by the drug. Also, include more caveats and in the discussion define more clearly the limitations of the study. Given the negative result of your study, it is also recommended that in the discussion you point to future experiments involving striatum and parietal cortex that may reveal

2. Please provide the information about the duration of the recording sessions and address a possibility that the effects of the drug may have changed with time after the ingestion of the drug. Reviewer 2 suggests that you assess this possibility by splitting the behavioral data into early and late.

3. Considering the difference in the behavioral effects between the two animals, please address the point raised by Reviewer 2 concerning the difference in weight between the two animals and the resulting difference in the dose of MPH administered to them.

4. Please provide the statistic to support the claim that there was no difference in the type of errors the animals made

5. Please address the comments from Reviewer 1 concerning receptor types, the terms “attention” and “saccade plan” and fix the legend for Fig 3.

--------

Reviewer 1

This paper addresses the important and understudied effects of methylphenidate, which is widely given to children, on neural activity in prefrontal cortex. They recorded a large sample of neural activity and administered a wide range of drug doses. I think this is an important null result to publish.

## References

[B1] Aarts E, Verhage M, Veenvliet JV, Dolan CV, van der Sluis S (2014) A solution to dependency: using multilevel analysis to accommodate nested data. Nat Neurosci 17:491–496. 10.1038/nn.3648 24671065

[B2] Acker L, Pino EN, Boyden ES, Desimone R (2016) FEF inactivation with improved optogenetic methods. Proc Acad Natl Sci USA 113:E7297–E7306. 10.1073/pnas.1610784113 PMC5135345 27807140

[B3] Armstrong KM, Chang MH, Moore T (2009) Selection and maintenance of spatial information by frontal eye field neurons. J Neurosci 29:15621–15629. 10.1523/JNEUROSCI.4465-09.2009 20016076 PMC3351279

[B4] Arnsten AF, Dudley AG (2005) Methylphenidate improves prefrontal cortical cognitive function through alpha2 adrenoceptor and dopamine D1 receptor actions: relevance to therapeutic effects in attention deficit hyperactivity disorder. Behav Brain Funct 1:2. 10.1186/1744-9081-1-2 15916700 PMC1143775

[B5] Averbeck BB, Latham PE, Pouget A (2006) Neural correlations, population coding and computation. Nat Rev Neurosci 7:358–366. 10.1038/nrn1888 16760916

[B6] Bain JN, Prendergast MA, Terry AV, Arneric SP, Smith MA, Buccafusco JJ (2003) Enhanced attention in rhesus monkeys as a common factor for the cognitive effects of drugs with abuse potential. Psychopharmacology (Berl) 169:150–160. 10.1007/s00213-003-1483-1 12768267

[B7] Barbas H, Pandya DN (1989) Architecture and intrinsic connections of the prefrontal cortex in the rhesus monkey. J Comp Neurol 286:353–375. 10.1002/cne.902860306 2768563

[B8] Berridge CW, Arnsten AF (2015) Catecholamine mechanisms in the prefrontal cortex: proven strategies for enhancing higher cognitive function. Curr Opin Behav Sci 4:33–40. 10.1016/j.cobeha.2015.01.002

[B9] Berridge CW, Devilbiss DM, Andrzejewski ME, Arnsten AFT, Kelley AE, Schmeichel B, Hamilton C, Spencer RC (2006) Methylphenidate preferentially increases catecholamine neurotransmission within the prefrontal cortex at low doses that enhance cognitive function. Biol Psychiatry 60:1111–1120. 10.1016/j.biopsych.2006.04.022 16806100

[B10] Berridge CW, Shumsky JS, Andrzejewski ME, McGaughy JA, Spencer RC, Devilbiss DM, Waterhouse BD (2012) Differential sensitivity to psychostimulants across prefrontal cognitive tasks: differential involvement of noradrenergic α_1_ - and α_2_-receptors. Biol Psychiatry 71:467–473. 10.1016/j.biopsych.2011.07.022 21890109 PMC3233638

[B11] Buschman TJ, Miller EK (2007) Top-down versus bottom-up control of attention in the prefrontal and posterior parietal cortices. Science 315:1860–1862. 10.1126/science.1138071 17395832

[B12] Buzsáki G (2004) Large-scale recording of neuronal ensembles. Nat Neurosci 7:446–451. 10.1038/nn1233 15114356

[B13] Chang CC, Lin CJ (2011) LIBSVM: a library for support vector machines. ACM Trans Intell Syst Technol (TIST) 2:27 10.1145/1961189.1961199

[B14] Clark K, Squire RF, Merrikhi Y, Noudoost B (2015) Visual attention: linking prefrontal sources to neuronal and behavioral correlates. Prog Neurobiol 132:59–80. 10.1016/j.pneurobio.2015.06.006 26159708

[B15] Cohen J (1992) A power primer. Psychol Bull 112:155–159. 10.1037/0033-2909.112.1.155 19565683

[B16] Cohen MR, Maunsell JHR (2009) Attention improves performance primarily by reducing interneuronal correlations. Nat Neurosci 12:1594–1600. 10.1038/nn.2439 19915566 PMC2820564

[B17] Cohen MR, Kohn A (2011) Measuring and interpreting neuronal correlations. Nat Neurosci 14:811–819. 10.1038/nn.2842 21709677 PMC3586814

[B18] Curtis CE, D'Esposito M (2004) The effects of prefrontal lesions on working memory performance and theory. Cogn Affect Behav Neurosci 4:528–539. 10.3758/CABN.4.4.528 15849895

[B19] Desimone R, Duncan J (1995) Neural mechanisms of selective visual attention. Annu Rev Neurosci 18:193–222. 10.1146/annurev.ne.18.030195.001205 7605061

[B20] Devilbiss DM, Berridge CW (2008) Cognition-enhancing doses of methylphenidate preferentially increase prefrontal cortex neuronal responsiveness. Biol Psychiatry 64:626–635. 10.1016/j.biopsych.2008.04.037 18585681 PMC2603602

[B21] Dias EC, Segraves MA (1999) Muscimol-induced inactivation of monkey frontal eye field: effects on visually and memory-guided saccades. J Neurophysiol 81:2191–2214. 10.1152/jn.1999.81.5.2191 10322059

[B22] Dockree PM, Barnes JJ, Matthews N, Dean AJ, Abe R, Nandam LS, Kelly SP, Bellgrove MA, O’Connell RG (2017) The effects of methylphenidate on the neural signatures of sustained attention. Biol Psychiatry 82:687–694. 10.1016/j.biopsych.2017.04.016 28599833

[B23] Doerge DR, Fogle CM, Paule MG, McCullagh M, Bajic S (2000) Analysis of methylphenidate and its metabolite ritalinic acid in monkey plasma by liquid chromatography/electrospray ionization mass spectrometry. Rapid Commun Mass Sp 14:619–623. 10.1002/(SICI)1097-0231(20000430)14:8<619::AID-RCM916>3.0.CO;2-2 10786896

[B24] Gamo NJ, Wang M, Arnsten AFT (2010) Methylphenidate and atomoxetine enhance prefrontal function through α2-adrenergic and dopamine D1 receptors. J Am Acad Child Adolesc Psychiatry 49:1011–1023. 10.1016/j.jaac.2010.06.015 20855046 PMC2999884

[B25] Goldman-Rakic PS (1995) Cellular basis of working memory. Neuron 14:477–485. 10.1016/0896-6273(95)90304-6 7695894

[B26] Gregoriou GG, Gotts SJ, Zhou H, Desimone R (2009) High-frequency, long-range coupling between prefrontal and visual cortex during attention. Science 324:1207–1210. 10.1126/science.1171402 19478185 PMC2849291

[B27] Gregoriou GG, Gotts SJ, Desimone R (2012) Cell-type-specific synchronization of neural activity in FEF with V4 during attention. Neuron 73:581–594. 10.1016/j.neuron.2011.12.019 22325208 PMC3297082

[B28] Hannestad J, Gallezot J-D, Planeta-Wilson B, Lin S-F, Williams WA, van Dyck CH, Malison RT, Carson RE, Ding Y-S (2010) Clinically relevant doses of methylphenidate significantly occupy norepinephrine transporters in humans in vivo. Biol Psychiatry 68:854–860. 10.1016/j.biopsych.2010.06.017 20691429 PMC3742016

[B29] Hebb DO (1949) The organization of behavior. New York: Wiley & Sons.

[B30] Ilieva IP, Hook CJ, Farah MJ (2015) Prescription stimulants’ effects on healthy inhibitory control, working memory, and episodic memory: a meta-analysis. J Cogn Neurosci 27:1069–1089. 10.1162/jocn_a_00776 25591060

[B31] Kanitscheider I, Coen-Cagli R, Pouget A (2015) Origin of information-limiting noise correlations. Proc Acad Natl Sci USA 112:E6973–E6982. 10.1073/pnas.1508738112 PMC4687541 26621747

[B32] Kimko HC, Cross JT, Abernethy DR (1999) Pharmacokinetics and clinical effectiveness of methylphenidate. Clin Pharmacokinet 37:457–470. 10.2165/00003088-199937060-00002 10628897

[B33] Kodama T, Kojima T, Honda Y, Hosokawa T, Tsutsui K-I, Watanabe M (2017) Oral administration of methylphenidate (ritalin) affects dopamine release differentially between the prefrontal cortex and striatum: a microdialysis study in the monkey. J Neurosci 37:2387–2394. 10.1523/JNEUROSCI.2155-16.2017 28154152 PMC6596846

[B34] Leavitt ML, Pieper F, Sachs A, Joober R, Martinez-Trujillo JC (2013) Structure of spike count correlations reveals functional interactions between neurons in dorsolateral prefrontal cortex area 8a of behaving primates. PLoS One 8:e61503. 10.1371/journal.pone.0061503 23630595 PMC3632589

[B35] Leavitt ML, Mendoza-Halliday D, Martinez-Trujillo JC (2017a) Sustained activity encoding working memories: not fully distributed. Trends Neurosci 40:328–346. 10.1016/j.tins.2017.04.004 28515011

[B36] Leavitt ML, Pieper F, Sachs AJ, Martinez-Trujillo JC (2017b) Correlated variability modifies working memory fidelity in primate prefrontal neuronal ensembles. Proc Acad Natl Sci USA 114:E2494–E2503. 10.1073/pnas.1619949114 28275096 PMC5373382

[B37] Lennert T, Martinez-Trujillo J (2011) Strength of response suppression to distracter stimuli determines attentional-filtering performance in primate prefrontal neurons. Neuron 70:141–152. 10.1016/j.neuron.2011.02.041 21482363

[B38] Lennert T, Cipriani R, Jolicoeur P, Cheyne D, Martinez-Trujillo JC (2011) Attentional modulation of neuromagnetic evoked responses in early human visual cortex and parietal lobe following a rank-order rule. J Neurosci 31:17622–17636. 10.1523/JNEUROSCI.4781-11.2011 22131423 PMC6623792

[B39] Lin M, Lucas HC Jr, Shmueli G (2013) Research commentary—too big to fail: large samples and the p-value problem. Inf Syst 24:906–917. 10.1287/isre.2013.0480

[B40] Mehta MA, Owen AM, Sahakian BJ, Mavaddat N, Pickard JD, Robbins TW (2000) Methylphenidate enhances working memory by modulating discrete frontal and parietal lobe regions in the human brain. J Neurosci 20:RC65. 10.1523/JNEUROSCI.20-06-j0004.2000 10704519 PMC6772505

[B41] Miller EK, Cohen JD (2001) An integrative theory of prefrontal cortex function. Annu Rev Neurosci 24:167–202. 10.1146/annurev.neuro.24.1.167 11283309

[B42] Mitchell JF, Sundberg KA, Reynolds JH (2009) Spatial attention decorrelates intrinsic activity fluctuations in macaque area V4. Neuron 63:879–888. 10.1016/j.neuron.2009.09.013 19778515 PMC2765230

[B43] Moore T, Fallah M (2004) Microstimulation of the frontal eye field and its effects on covert spatial attention. J Neurophysiol 91:152–162. 10.1152/jn.00741.2002 13679398

[B44] Moran J, Desimone R (1985) Selective attention gates visual processing in the extrastriate cortex. Science 229:782–784. 10.1126/science.4023713 4023713

[B45] Moreno-Bote R, Beck J, Kanitscheider I, Pitkow X, Latham P, Pouget A (2014) Information-limiting correlations. Nat Neurosci 17:1410–1417. 10.1038/nn.3807 25195105 PMC4486057

[B46] Nicolelis MA, Fanselow EE, Ghazanfar AA (1997) Hebb's dream: the resurgence of cell assemblies. Neuron 19:219–221. 10.1016/S0896-6273(00)80932-0 9292712

[B47] Nicolelis MAL, Dimitrov D, Carmena JM, Crist R, Lehew G, Kralik JD, Wise SP (2003) Chronic, multisite, multielectrode recordings in macaque monkeys. Proc Natl Acad Sci USA 100:11041–11046. 10.1073/pnas.1934665100 12960378 PMC196923

[B48] Niebergall R, Khayat PS, Treue S, Martinez-Trujillo JC (2011) Multifocal attention filters targets from distracters within and beyond primate MT neurons' receptive field boundaries. Neuron 72:1067–1079. 10.1016/j.neuron.2011.10.013 22196340

[B49] Noudoost B, Moore T (2011) Control of visual cortical signals by prefrontal dopamine. Nature 474:372–375. 10.1038/nature09995 21572439 PMC3117113

[B50] Palomero-Gallagher N, Zilles K (2004) Isocortex In: The rat nervous system (Paxinos G, ed), Ed 6, pp 729–757. San Diego: Elsevier.

[B51] Petersen SE, Posner MI (2012) The attention system of the human brain: 20 years after. Annu Rev Neurosci 35:73–89. 10.1146/annurev-neuro-062111-150525 22524787 PMC3413263

[B52] Peterson BS, Potenza MN, Wang Z, Zhu H, Martin A, Marsh R, Plessen KJ, Yu S (2009) An FMRI study of the effects of psychostimulants on default-mode processing during Stroop task performance in youths with ADHD. Am J Psychiatry 166:1286–1294. 10.1176/appi.ajp.2009.08050724 19755575 PMC3289412

[B53] Petrides M (2005) Lateral prefrontal cortex: architectonic and functional organization. Philos Trans R Soc Lond B Biol Sci 360:781–795. 10.1098/rstb.2005.1631 15937012 PMC1569489

[B54] Petrides M, Pandya DN (1994) Comparative architectonic analysis of the human and the macaque frontal cortex In: Handbook of neuropsychology (Grafman J, Boller F, eds), pp 17–58. Amsterdam: Elsevier.

[B55] Petrides M, Pandya DN (1999) Dorsolateral prefrontal cortex: comparative cytoarchitectonic analysis in the human and the macaque brain and corticocortical connection patterns. Eur J Neurosci 11:1011–1036. 10.1046/j.1460-9568.1999.00518.x 10103094

[B56] Petrides M, Pandya DN (2006) Efferent association pathways originating in the caudal prefrontal cortex in the macaque monkey. J Comp Neurol 498:227–251. 10.1002/cne.21048 16856142

[B57] Prendergast MA, Jackson WJ, Terry AV, Kille NJ, Arneric SP, Decker MW, Buccafusco JJ (1998) Age-related differences in distractibility and response to methylphenidate in monkeys. Cereb Cortex 8:164–172. 10.1093/cercor/8.2.164 9542895

[B58] Preuss TM, Goldman-Rakic PS (1991) Myelo- and cytoarchitecture of the granular frontal cortex and surrounding regions in the strepsirhine primate Galago and the anthropoid primate Macaca. J Comp Neurol 310:429–474. 10.1002/cne.903100402 1939732

[B59] Quian Quiroga R, Panzeri S (2009) Extracting information from neuronal populations: information theory and decoding approaches. Nat Rev Neurosci 10:173–185. 10.1038/nrn2578 19229240

[B60] Rajala AZ, Henriques JB, Populin LC (2012) Dissociative effects of methylphenidate in nonhuman primates: trade-offs between cognitive and behavioral performance. J Cogn Neurosci 24:1371–1381. 10.1162/jocn_a_00225 22401288 PMC3409297

[B61] Reynolds JH, Chelazzi L (2004) Attentional modulation of visual processing. Annu Rev Neurosci 27:611–647. 10.1146/annurev.neuro.26.041002.131039 15217345

[B62] Rosa-Neto P, Lou HC, Cumming P, Pryds O, Karrebaek H, Lunding J, Gjedde A (2005) Methylphenidate-evoked changes in striatal dopamine correlate with inattention and impulsivity in adolescents with attention deficit hyperactivity disorder. Neuroimage 25:868–876. 10.1016/j.neuroimage.2004.11.031 15808987

[B63] Rudebeck PH, Murray EA (2011) Dissociable effects of subtotal lesions within the macaque orbital prefrontal cortex on reward-guided behavior. J Neurosci 31:10569–10578. 10.1523/JNEUROSCI.0091-11.2011 21775601 PMC3171204

[B64] Rudebeck PH, Saunders RC, Prescott AT, Chau LS, Murray EA (2013) Prefrontal mechanisms of behavioral flexibility, emotion regulation and value updating. Nat Neurosci 16:1140–1145. 10.1038/nn.3440 23792944 PMC3733248

[B65] Schafer RJ, Moore T (2011) Selective attention from voluntary control of neurons in prefrontal cortex. Science 332:1568–1571. 10.1126/science.1199892 21617042 PMC3371378

[B66] Shadlen MN, Britten KH, Newsome WT, Movshon JA (1996) A computational analysis of the relationship between neuronal and behavioral responses to visual motion. J Neurosci 16:1486–1510. 10.1523/JNEUROSCI.16-04-01486.1996 8778300 PMC6578557

[B67] Simmons JM, Minamimoto T, Murray EA, Richmond BJ (2010) Selective ablations reveal that orbital and lateral prefrontal cortex play different roles in estimating predicted reward value. J Neurosci 30:15878–15887. 10.1523/JNEUROSCI.1802-10.2010 21106826 PMC3021956

[B68] Soto PL, Dallery J, Ator NA, Katz BR (2013) A critical examination of best dose analysis for determining cognitive-enhancing potential of drugs: studies with rhesus monkeys and computer simulations. Psychopharmacology (Berl) 228:611–622. 10.1007/s00213-013-3070-4 23529381 PMC3729620

[B69] Spencer RC, Klein RM, Berridge CW (2012) Psychostimulants act within the prefrontal cortex to improve cognitive function. Biol Psychiatry 72:221–227. 10.1016/j.biopsych.2011.12.002 22209638 PMC3319517

[B70] Spencer RC, Devilbiss DM, Berridge CW (2015) The cognition-enhancing effects of psychostimulants involve direct action in the prefrontal cortex. Biol Psychiatry 77:940–950. 10.1016/j.biopsych.2014.09.013 25499957 PMC4377121

[B71] Squire RF, Noudoost B, Schafer RJ, Moore T (2013) Prefrontal contributions to visual selective attention. Annu Rev Neurosci 36:451–466. 10.1146/annurev-neuro-062111-150439 23841841

[B72] Subcommittee on Attention-Deficit/Hyperactivity Disorder, Steering Committee on Quality Improvement and Management, Wolraich M, Brown L, Brown RT, DuPaul G, Earls M, Feldman HM, Ganiats TG, Kaplanek B, Meyer B, Perrin J, Pierce K, Reiff M, Stein MT, Visser S (2011) ADHD: clinical practice guideline for the diagnosis, evaluation, and treatment of attention-deficit/hyperactivity disorder in children and adolescents. Pediatrics 128:1007–1022. 10.1542/peds.2011-2654 22003063 PMC4500647

[B73] Tomasi D, Volkow ND, Wang GJ, Wang R, Telang F, Caparelli EC, Wong C, Jayne M, Fowler JS (2011) Methylphenidate enhances brain activation and deactivation responses to visual attention and working memory tasks in healthy controls. Neuroimage 54:3101–3110. 10.1016/j.neuroimage.2010.10.060 21029780 PMC3020254

[B74] Tremblay S, Doucet G, Pieper F, Sachs A, Martinez-Trujillo J (2015a) Single-trial decoding of visual attention from local field potentials in the primate lateral prefrontal cortex is frequency-dependent. J Neurosci 35:9038–9049. 10.1523/JNEUROSCI.1041-15.2015 26085629 PMC6605161

[B75] Tremblay S, Pieper F, Sachs A, Martinez-Trujillo J (2015b) Attentional filtering of visual information by neuronal ensembles in the primate lateral prefrontal cortex. Neuron 85:202–215. 10.1016/j.neuron.2014.11.021 25500502

[B76] Treue S, Martínez Trujillo JC (1999) Feature-based attention influences motion processing gain in macaque visual cortex. Nature 399:575–579. 10.1038/21176 10376597

[B77] Visser SN, Danielson ML, Bitsko RH, Holbrook JR, Kogan MD, Ghandour RM, Perou R, Blumberg SJ (2014) Trends in the parent-report of health care provider-diagnosed and medicated attention-deficit/hyperactivity disorder: United States, 2003-2011. J Am Acad Child Adolesc Psychiatry 53:34–46.e2. 10.1016/j.jaac.2013.09.001 24342384 PMC4473855

[B78] Volkow ND, Ding YS, Fowler JS, Wang GJ, Logan J, Gatley JS, Dewey S, Ashby C, Liebermann J, Hitzemann R, Wolf AP (1995) Is methylphenidate like cocaine? Studies on their pharmacokinetics and distribution in the human brain. Arch Gen Psychiatry 52:456–463. 10.1001/archpsyc.1995.03950180042006 7771915

[B79] Volkow ND, Wang GJ, Fowler JS, Gatley SJ, Logan J, Ding YS, Hitzemann R, Pappas N (1998) Dopamine transporter occupancies in the human brain induced by therapeutic doses of oral methylphenidate. Am J Psychiatry 155:1325–1331. 10.1176/ajp.155.10.1325 9766762

[B80] Volkow ND, Wang GJ, Fowler JS, Ding YS (2005) Imaging the effects of methylphenidate on brain dopamine: new model on its therapeutic actions for attention-deficit/hyperactivity disorder. Biol Psychiatry 57:1410–1415. 10.1016/j.biopsych.2004.11.006 15950015

[B83] Wargin W, Patrick K, Kilts C, Gualtieri CT, Ellington K, Mueller RA, Kraemer G, Breese GR (1983) Pharmacokinetics of methylphenidate in man, rat and monkey. J. Pharmacol. Exp. Ther 226:382–386. 6410043

[B81] Wasserstein RL, Lazar NA (2016) The ASA’s statement on p-values: context, process, and purpose. Am Stat 70:129–133. 10.1080/00031305.2016.1154108

[B82] Winstanley CA, Eagle DM, Robbins TW (2006) Behavioral models of impulsivity in relation to ADHD: translation between clinical and preclinical studies. Clin Psychol Rev 26:379–395. 10.1016/j.cpr.2006.01.001 16504359 PMC1892795

